# Supplementary material for: Directed Evolution of 4-Hydroxyphenylpyruvate Biosensors Based on a Dual Selection System
Source: Int J Mol Sci. 2024 Jan 26;25(3):1533. doi: 10.3390/ijms25031533 (PMC10855707; doi:10.3390/ijms25031533)
Supplement: Supplementary file 1 [file ijms-25-01533-s001.zip › ijms-2798644-supplementary.pdf]

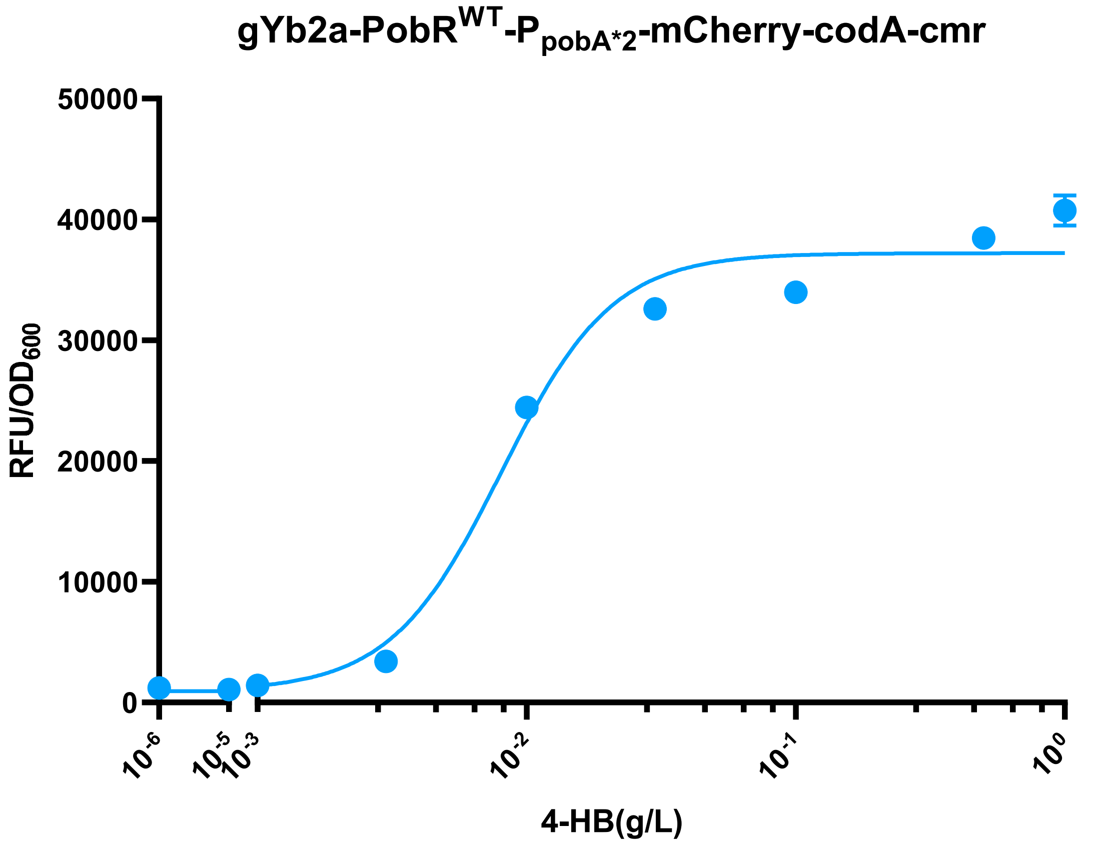

Supplementary Figure S1. The dynamic range of the PobR^{WT} Biosensor (in *E. coli* BWΔ*codA*) responsive to 4HB. The vertical axis is the ratio of the reporter gene *mCherry* (RFU) expression to the growth of *E. coli* (OD_{600}), measured after a 12 h of cultivation in M9 medium. Each value represents the mean ± standard deviation from 3 biological replicates.

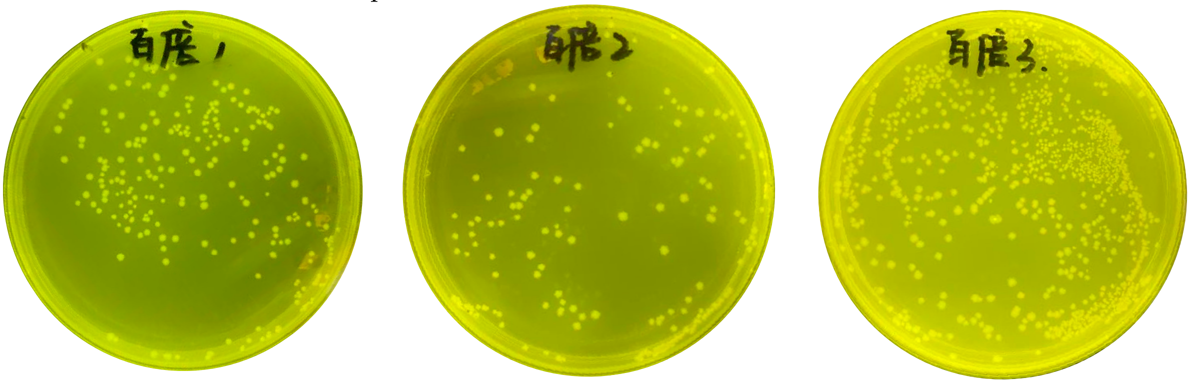

Supplementary Figure S2. Clone calculation after passing two rounds of negative selection in medium containing 50 mg/L and 200 mg/L 5-FC using the dilution coating method. The experimental procedure includes aspirating 50 μl of the selected bacterial solution, adding it into 5000 μl of LB medium (i.e., 1:100 dilution), and then taking 50 μl of the diluted bacteria to spread onto a plate. Based on our calculation, the original density of the two-round selected bacteria was 450,000 CFU/mL (The three different black markings on the plates

represent three different biological replicates for colony counting at 1:100 dilution).

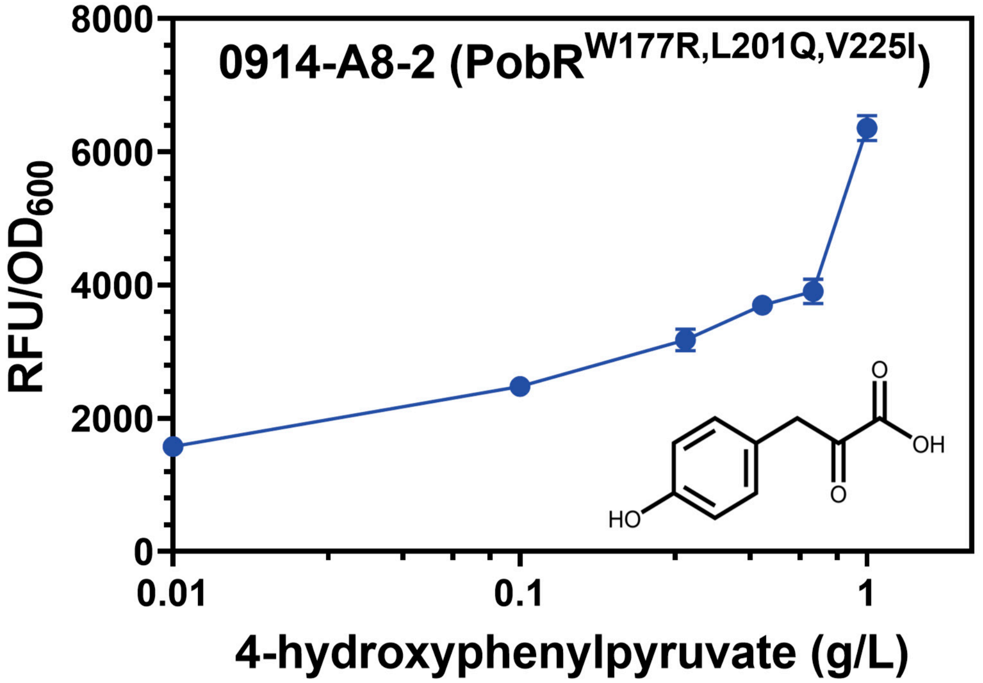

Supplementary Figure S3. Range curve of PobR$^{W177R,\ L201Q,\ V225I}$ response to HPP. The vertical axis is the ratio of reporter gene mCherry (RFU) expression to E. coli (OD 600) growth measured after 12 hours of culture in M9 medium with different concentrations of HPP. Each value represents the mean ± standard deviation of 3 biological replicates.

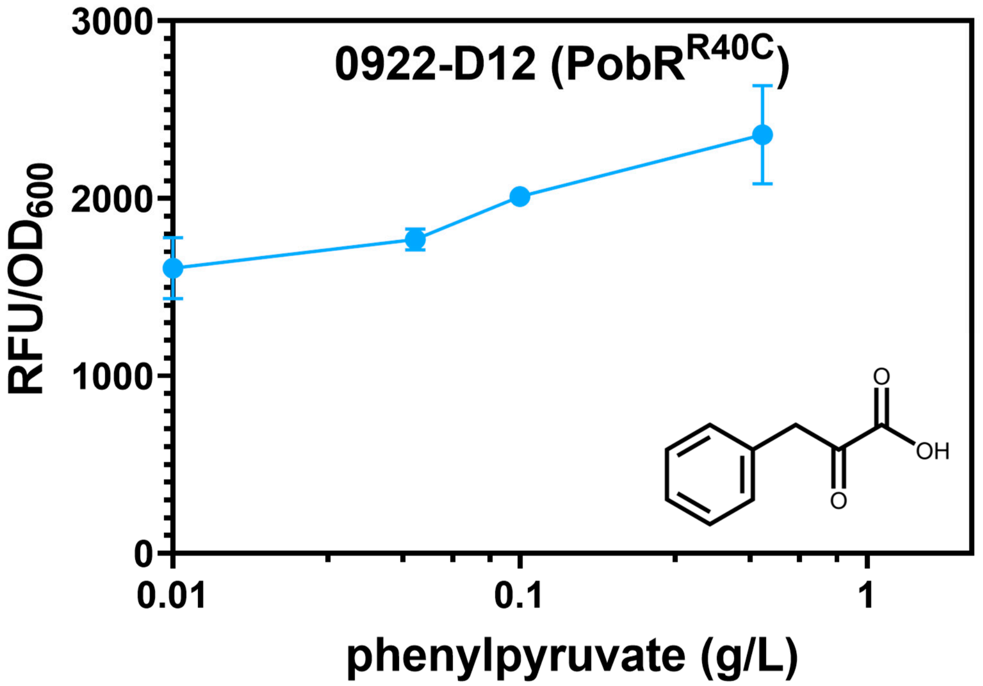

Supplementary Figure S4. Range curve of PobR^{R40C} response to PPA. The vertical axis is the ratio of reporter gene mCherry (RFU)

expression to E. coli (OD 600) growth measured after 12 hours of culture in M9 medium with different concentrations of PPA. Each value represents the mean ± standard deviation of 3 biological replicates.

a

Hydrophobic Interactions

Index	Residue	AA	Distance	Ligand Atom	Protein Atom
1	155A	PRO	3.62	2149	726
2	155A	PRO	3.80	2150	727
3	184A	LYS	3.80	2153	1087

Hydrogen Bonds

Index	Residue	AA	Distance H-A	Distance D-A	Donor Angle	Protein donor?	Side chain	Donor Atom	Acceptor Atom
1	126A	GLU	2.07	2.90	146.79	√	√	372 [O3]	2159 [O3]
2	184A	LYS	2.21	3.20	164.97	√	×	1081 [Nam]	2158 [O3]

Salt Bridges

Index	Residue	AA	Distance	Protein positive?	Ligand Group	Ligand Atoms
1	125A	HIS	5.16	√	Carboxylate	2156, 2157
2	157A	HIS	5.01	√	Carboxylate	2156, 2157

b

Hydrophobic Interactions

Index	Residue	AA	Distance	Ligand Atom	Protein Atom
1	125A	HIS	3.71	2142	353
2	155A	PRO	3.98	2144	726
3	157A	HIS	3.96	2138	747
4	166A	LEU	3.48	2139	853
5	177A	ARG	3.28	2139	982
6	181A	TYR	3.74	2137	1039
7	183A	LEU	3.74	2138	1065

Hydrogen Bonds

Index	Residue	AA	Distance H-A	Distance D-A	Donor Angle	Protein donor?	Side chain	Donor Atom	Acceptor Atom
1	157A	HIS	1.94	2.92	162.13	√	×	743 [Nam]	2145 [O.co2]

Salt Bridges

Index	Residue	AA	Distance	Protein positive?	Ligand Group	Ligand Atoms
1	157A	HIS	4.09	√	Carboxylate	2145, 2146

Supplementary Figure S5. PLIP was used to analyze the non-covalent interactions of PobR protein with ligands. (a) The PobR^{WT}

protein has three hydrophobic interactions, two hydrogen bonds and two salt bridges with HPP. (b) The PobR^{W177R} has seven hydrophobic interactions, one hydrogen bonds and one salt bridges with HPP.

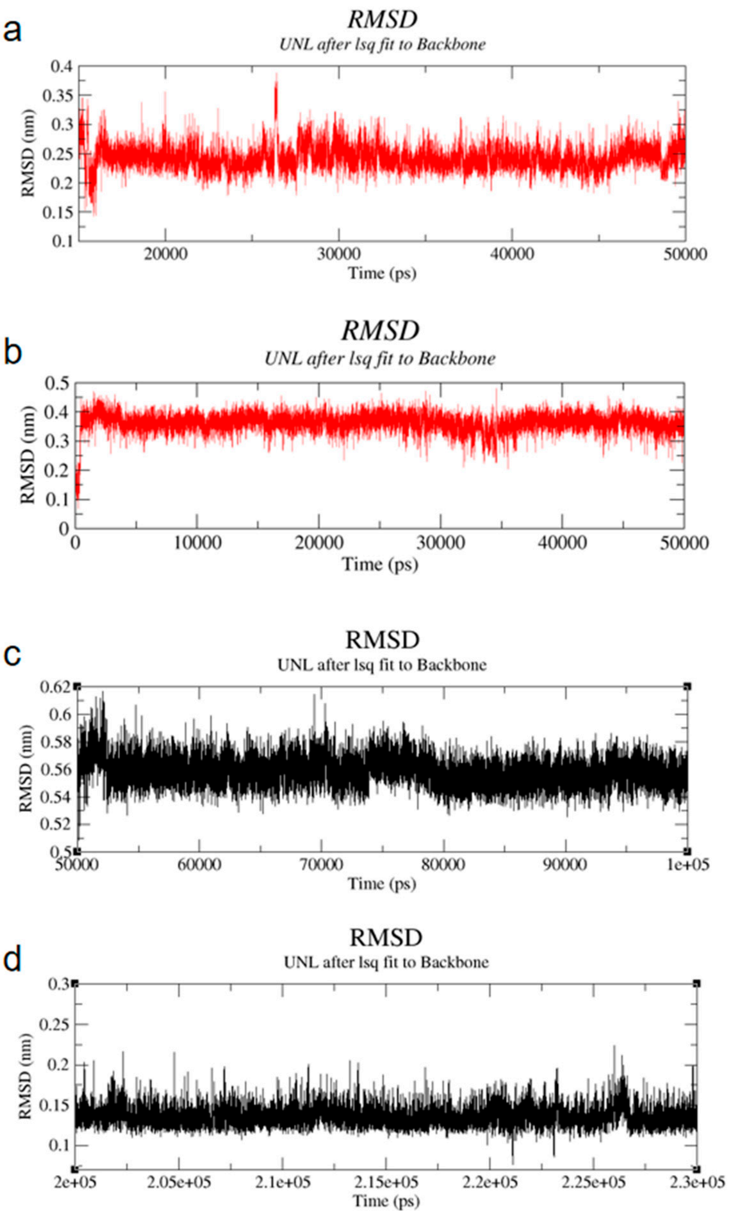

Supplementary Figure S6. The stability of the system was preliminarily measured by RMSD curve. (a) The RMSD curve of PobR^{WT} to 4HB. (b) The RMSD curve of PobR^{W177R} to 4HB. (c) The RMSD curve of Pob^{RWT} to HPP. (d) The RMSD curve of PobR^{W177R} to HPP.

Supplementary Table S1. Bacterial strains and plasmids used in this study.

Strain	Description	Source
DB3.1	*F- gyrA462 endA1 glnV44 Δ(sr1-recA) mcrB mrr hsdS20(rB-, mB-) ara14 galK2 lacY1 proA2 rpsL20 (SmR) xyl5 Δleumtl1*	Lab stock
BW25113Δ*codA*	rrnBT14 Δ*lacZWJ16* hsdR514 Δ*araBAD*$_{AH33}$ Δ*rhaBAD*$_{LD78}$Δ*codA*	Lab stock

plasmid	Description	Source
gYB2a-ccdb	*Amp*^{r}, P15A ori, *ccdb* gene	Lab stock
pYB1a-eGFP-cmr	*Amp*^{r}, P15A ori, *eGFP* gene, *Chl*^{r}	Lab stock
pUAM-RE-CD	*Amp*^{r}, Anderson J23100-promter, *RE* gene, *codA* gene	Lab stock
pYP1a- PobR-P$_{pobA}$*2- mCherry-sacB	*Amp*^{r}, P15A ori,two P$_{pobA}$ promotor, *pobR* gene, *mCherry* gene, *sacB* gene	Lab stock
gYB2a-P$_{pobA}$*2-mCherry-sacB	*Amp*^{r}, P15A ori, two P$_{pobA}$ promotors, *mCherry* gene, *sacB* gene	this study
gYb2a-P$_{pobA}$*2-mCherry-SacB-cmr	*Amp*^{r}, P15A ori,two P$_{pobA}$ promotor, *mCherry* gene, *sacB* gene, *Chl*^{r}	this study
gYb2a-P$_{pobA}$*2-mCherry-CD-cmr	*Amp*^{r}, P15A ori,two P$_{pobA}$ promotor, *mCherry* gene, *codA* gene, *Chl*^{r}	this study
gYb2a-PobR-mCherry-CD-cmr	*Amp*^{r}, P15A ori,two P$_{pobA}$ promotor, *pobR* gene, *mCherry* gene, *codA* gene, *Chl*^{r}	this study

Supplementary Table S2. Primers used in this study.

Primers	Sequences (5′-3′)
PpobA*2-mc-0311-F	gagggtctctcatccgagacgggtaccATTGGTGATGCTGTTCCAT
Primer 2-0311-R	ggatctcctgctgtatgtcggaattcttatttgttaactgttaatt
cmr-gibson-0317-F	gatctcctgctgtatgtcggaattcttacgccccgccctgccact
cmr-gibson-0317-R	aattaacagttaacaaataagaattcatggagaaaaaaatcactgg
CD-gibson-0420-R	ggtatatccagtgatttttttctccatgaattcttaccgtttgtaatcgatgg
CD-gibson-0420-F	gtaactcgagaggagatgtcgaataacgctttac
cmr-Gibson-F	caaacggtaagaattcatggagaaaaaaatcactggatatacc
Mc-Gibson-R	gacatctcctctcgagttacttgtacagctcgtccatg
PobR-P1-BsaI-F	gtgctgGGTCTCggATGGAACAGCATCACCAATA
PobR-P2-BsaI-R	agcgtgGGTCTCTGCTAAACCAAGTTGCGCAGTTCAT
